# Sex hormones influence the intestinal microbiota composition in mice

**DOI:** 10.3389/fmicb.2022.964847

**Published:** 2022-10-31

**Authors:** Yi Wu, Xinxin Peng, Xiaoya Li, Dandan Li, Zhoujin Tan, Rong Yu

**Affiliations:** ^1^College of Chinese Medicine, Hunan University of Chinese Medicine, Changsha, China; ^2^Hunan Key Laboratory of Chinese Medicine Prescription and Syndromes Translational Medicine, Changsha, China; ^3^Department of Pediatrics, The First Affiliated Hospital of Hunan University of Chinese Medicine, Changsha, China

**Keywords:** intestinal mucosa-associated microbiota, intestinal luminal content microbiota, sex hormones, sex dimorphism traits, gender-associated diseases

## Abstract

Sex hormone secretion difference is one of the main reasons for sexually dimorphic traits in animals, which affects the dimorphism of the intestinal microbiota; however, their interaction is still unknown. Intestinal mucosa-associated microbiota (MAM) and intestinal luminal content microbiota (LM) belong to two different habitats according to the difference in interactions between bacteria and host intestinal epithelium/nutrients. To clarify the sexually dimorphic characteristics of MAM and LM and their correlation with sex hormones, 12 specific pathogen-free (SPF) Kunming mice from the same nest were fed separately according to sex. After 8 weeks, samples from the male intestinal mucosa group (MM group), the female intestinal mucosa group (FM group), the male intestinal content group (MC group), and the female intestinal content group (FC group) were collected and then, the next-generation sequencing of 16S ribosomal ribonucleic acid (rRNA) gene was performed. Our results showed that the sexual dimorphism of MAM was more obvious than that of LM and the relative abundance of *Muribaculaceae, Turicibacter*, and *Parasutterella* was significantly higher in the FM group than in the MM group (*p* < 0.001, *p* < 0.05, *p* < 0.05). Next, we measured the level of serum sex hormones in mice and calculated the correlation coefficient between major bacteria and sex hormones. The results showed that the correlation between MAM and sex hormones was more prominent, and finally, three bacterial genera (*Muribaculaceae, Turicibacter*, and *Parasutterella*) were obtained, which could better represent the relationship between sexual dimorphism and sex hormones. The abundance of *Parasutterella* is positively and negatively correlated with estradiol and testosterone (T), respectively, which may be related to the differences in the metabolism of bile acid and glucose. A decrease in the abundance of *Turicibacter* is closely related to autism. Our results show that the abundance of *Turicibacter* is negatively and positively correlated with T and estradiol, respectively, which can provide a hint for the prevalence of male autism. In conclusion, it is proposed in our study that intestinal microbiota is probably the biological basis of physiological and pathological differences due to sex, and intestinal MAM can better represent the sexual dimorphism of mice.

## Introduction

Significant differences in mammalian health and disease exist between males and females, that is, sexual dimorphism (Britannica, 2022). For example, in most mammalian species, males are slightly larger than females (Naqvi et al., 2019), and differences in longevity and aging processes have been observed between males and females (Sampathkumar et al., 2020). Sexually dimorphic traits are also evident in the incidence, epidemic, and mortality of diseases, such as autoimmune disorders and autism (Alshammari, 2021; Manuel and Liang, 2021). In recent years, gut microbiota has become a hotspot for various studies. Coincidentally, these studies found sex differences in gut microbiota composition between humans and rodents (Ding and Schloss, 2014; Falony et al., 2016; Borgo et al., 2018; Sinha et al., 2019). It has also been suggested that sex hormones play an important role in building and maintaining the characteristics of the gut microbiome associated with sex (Zhang X. et al., 2021).

Sexual dimorphism is controlled by sex hormones, which have a bidirectional interaction with the intestinal microbiota. On the one hand, sex hormones affect the intestinal microbiota by regulating the permeability and integrity of an intestinal barrier and adjusting sex hormone receptors, β-glucuronidase, bile acid, intestinal immunity, etc. (Braniste et al., 2009; Looijer-van Langen et al., 2011; Li and Chiang, 2015; Laffont et al., 2017; Pellock and Redinbo, 2017; Miranda-Ribera et al., 2019; Barroso et al., 2020). On the other hand, the intestinal microbiota also influences the secretion of sex hormones, for example, androgen. Testosterone (T) and ovaries are the major production sources of male and female androgen, respectively, and the intestinal microbiota is the major regulator of androgen metabolism in the intestinal tract (Pernigoni et al., 2021). Some bacterial strains have been shown to metabolize androgen *in vitro*. For example, *Aggregatibacter actinomycetecomitans* and *Porphyromonas gingivalis* convert T to dihydrotestosterone (Bélanger et al., 1989). Pathologically, abnormal fluctuations in androgen contribute to the development and progression of diseases by affecting the intestinal microbiota (Yurkovetskiy et al., 2013; Moreno-Indias et al., 2016). Female rats with pathological androgen levels were found to have different intestinal microbiota from normal rats. Studies showed that abnormal androgen levels can lead to intestinal dysbacteria, including enzymes involved in androgen metabolism, which further interfere with androgen metabolism, and are associated with diseases such as polycystic ovary syndrome (Lindheim et al., 2017; Liu et al., 2017; Torres et al., 2018; Zeng et al., 2018), type 1 diabetes (Markle et al., 2013; Yurkovetskiy et al., 2013), and obesity (Kelly and Jones, 2015; Harada et al., 2016). Therefore, intestinal microbiota dimorphism is also a part of sexual dimorphism, in which sex hormones play a crucial role.

In recent years, sufficient animal experiments and clinical trials have confirmed differences in composition and function between luminal content microbiota (LM) and mucosa-associated microbiota (MAM) (Van den Abbeele et al., 2011; Yang et al., 2020). MAM is believed to interact directly or indirectly with the host intestinal epithelium and is therefore critical to the formation of the host immune system. LM is mainly involved in the digestion of nutrients and does not interact directly with the intestinal mucosa. Therefore, the composition and function of MAM is closely related to the host and its immune system, while LM is closely related to nutrients (Van den Abbeele et al., 2011). Due to this difference, LM and MAM have different roles in the initiation and progression of diseases. MAM played the most important role in the pathogenesis of diarrhea-predominant irritable bowel syndrome (IBS-D) (Maharshak et al., 2018), MAM is highly susceptible to disruption in patients with diarrhea because it participates in neurological responses (Zhang C. et al., 2021). Functional MAM and LM differ from each other (He et al., 2019; Wu et al., 2020). According to Francesca Borgo, microbial diversity in these two niches might be influenced by host factors such as body mass index (BMI), diet, and sex. Thus, in our study, we controlled all variables except sex and focused on the effects of sex and sex hormones on intestinal microbiota diversity (Borgo et al., 2018).

In summary, we attempted to clarify the following questions: (i) Are host sex hormones involved in intestinal microbiota diversity? (ii) Is there a difference in the impact of host sex on MAM and LM diversity? (iii) Could the intestinal microbiota be one of the biological bases of sex-associated diseases?

## Materials and methods

### Materials

#### Animals and feeding

A total of 12 specific pathogen-free (SPF) Kunming mice (3 weeks of age, half male, half female) were purchased from Hunan Slike Jingda Laboratory Animal Co., Ltd. and fed in a regulated barrier system with light and dark cycles of 12 h, 23–25°C, and 50–70% relative humidity. After 1 week of adaptive feeding, animals were divided into male and female groups and fed for 8 weeks. Mice were fed by the Animal Experiment Center of Hunan University of Chinese Medicine with nutritional standards in line with GB/14924.3 and sanitation standards in line with GB/T149.24.2 to support their growth and reproduction. All animal experiments were licensed by the Animal Experiment of Hunan University of Chinese Medicine (Changsha, China), and the protocol was approved by the Animal Ethics Committee of Hunan University of Chinese Medicine [Facility use permit number: SYXK (Xiang) 2019-0009].

### Methods

#### Serum sex steroid-level testing

Post experiment, all mice were sacrificed by sampling orbital blood after fasting for 12 h; then, small intestinal contents and mucosa were collected according to the method established by our research group (Wu et al., 2021). Four serum sex hormones (estradiol, T, prolactin, and progesterone) were detected using chemiluminescence immunoassay. The operating instrument was the Abbott AXSYM automatic chemiluminescence instrument equipped with matching reagents. The nuclear medicine department of the First Affiliated Hospital of the Hunan University of Chinese Medicine is responsible for the detection. In the female group, intestinal contents were labeled as FC 1, FC 2, FC 3, FC 4, FC 5, and FC 6 and the intestinal mucosa was labeled as FM 1, FM 2, FM 3, FM 4, FM 5, and FM 6. In the male group, intestinal contents were labeled as MC 1, MC 2, MC 3, MC 4, MC 5, and MC 6 and the intestinal mucosa was labeled as MM 1, MM 2, MM 3, MM 4, MM 5, and MM 6. All samples were stored at −80°C for the high-throughput sequencing of the 16S ribosomal ribonucleic acid (rRNA) gene.

#### Extraction and polymerase chain reaction amplification of total DNA

The total microbial genomic deoxyribonucleic acid (DNA) from each sample was extracted as per the directives of the DNA extraction kit (MN NucleoSpin 96 So), and the steps were included as follows. The sample was precipitated to remove impurities and filtered to remove inhibitors, followed by DNA binding, membrane washing, drying, and elution. Quantity and mass of the extracted DNA were detected using NanoDrop ND-2000 ultramicro spectrophotometer (Thermo Fisher Scientific, Waltham, MA, USA) and Qubit 3.0 Fluorometer (Life Technologies, CA, USA), *via* agarose gel electrophoresis, respectively. All samples were processed by Beijing Biomac Biotechnology Co., Ltd. (Beijing, China).

#### Bridge polymerase chain reaction and 16S rRNA gene sequencing

Flow cells are the channels for adsorption of flowing DNA fragments. Adapter-added DNA fragments on a chip containing adapters are bound to flow cells and bridge-amplified. The primers, 338F5'-ACTCCTACGGGAGGCAGCA-3' and 806R5'-GGACTACHVGGGTWTCTAAT-3', were designed according to the conservative region of 16S r DNA V3-V4. The amplification reaction system was as follows: 50 ng of gene DNA, 0.3 μl of Vn F, 0.3 μl of Vn R, 5 μl of KOD FX Neo Buffer, 2 μl of deoxynucleoside triphosphate (dNTP), 0.25 μl of KOD FX Neo, and finally a total reaction volume to 10 μl of double pure water (ddH_2_O). Polymerase chain reaction (PCR) reaction conditions were given as follows: DNA was rapidly denatured at 95°C for 30 s and rapidly cooled to 50°C for 30 s, and the primers were annealed and bound to the target sequence and rapidly heated to 72°C for 40 s. After the last cycle, the primer strands were extended along the template for 7 min and were maintained at 4°C.

Library preparation and 16S rRNA gene sequencing were performed using the Solexa Genome Analyzer platform. When a complementary chain extends into a lateral column cluster family, each inserted dNTP can release corresponding fluorescence, which is immediately detected by a sequencer and then converted into fragment sequence information. Next-generation sequencing features a bridge polymerase chain reaction along with 16S rRNA gene sequencing. The basic principle is DNA polymerase and fluorescently labeled dNTPs and adapter primers in the amplification reaction system when a complementary chain extends into a lateral column cluster family.

#### Data processing

The raw sequencing data are processed with quality filtering, double-ended sequence splicing, and chimera elimination. The reads of each sample are spliced using the USEARCH (version 10) (Edgar Robert, 2013) with a minimum overlap length of 10 bp and a maximum mismatch ratio in the overlap area of 0.2 (Default), and the resulting splicing sequence is the Raw Tags. After quality inspection, tags with a length of less than 75% are filtered to get Clean Tags using the Trimmomatic program. To extract the final tag sequence, the chimera was eliminated using UCHIME (version 8.1) (Bokulich et al., 2013).

### Bioinformatics analysis and statistical methods

Sequences were clustered using USEARCH (version 10.0) (Edgar Robert, 2013) with a similarity criterion of 97% and the default OTU filtering threshold of 0.005% of all sequences. OTU (operational taxonomic units) were then aligned in the Silva database, and the species were annotated using the blast method. The α and β diversities were demonstrated by Chao 1, Shannon, ACE, Simpson index, non-metric multi-dimensional scaling (NMDS) analysis, and analysis of similarities (ANOSIM). The abundance of microbiota at all levels is calculated based on the OTU and is presented in a histogram. The random forest algorithm is an integrated algorithm that integrates multiple decision trees and can avoid the problem of overfitting a single decision tree. The Gini index calculates the influence of each variable on the observed heterogeneity at each node of the classification tree, and larger values indicate that the variables are more important. Our study uses line discriminant analysis effect size (LEfSe) and random forest algorithm analysis at the same time to find the biomarker for different groups. To further analyze the correlation between the intestinal microbiota and sex hormones, we calculated Spearman's rank correlation coefficient and plotted the heatmap. Compared to PICRUSt 1, PICRUSt 2 (Douglas et al., 2020) has the advantages of richer genomic information, more realistic prediction settings, and more rigorous functional prediction methods. The abovementioned analysis was performed using BMKCloud (www.biocloud.net).

Scatter and linear correlation plots (Pearson correlation method) were drawn using Graph pad Prism 9.0. Statistical data were analyzed using SPSS 24.0 software (IBM, Almonk, NY, USA). The independent sample *t*-test was used when the two groups of data were in agreement with the normal distribution. Otherwise, the nonparametric test (the Mann–Whitney *U* test) was used. The test criterion was a *p*-value < 0.05 or a *p*-value < 0.01.

## Results

### DNA sequence and the number of OTUs

**Figure 1:**
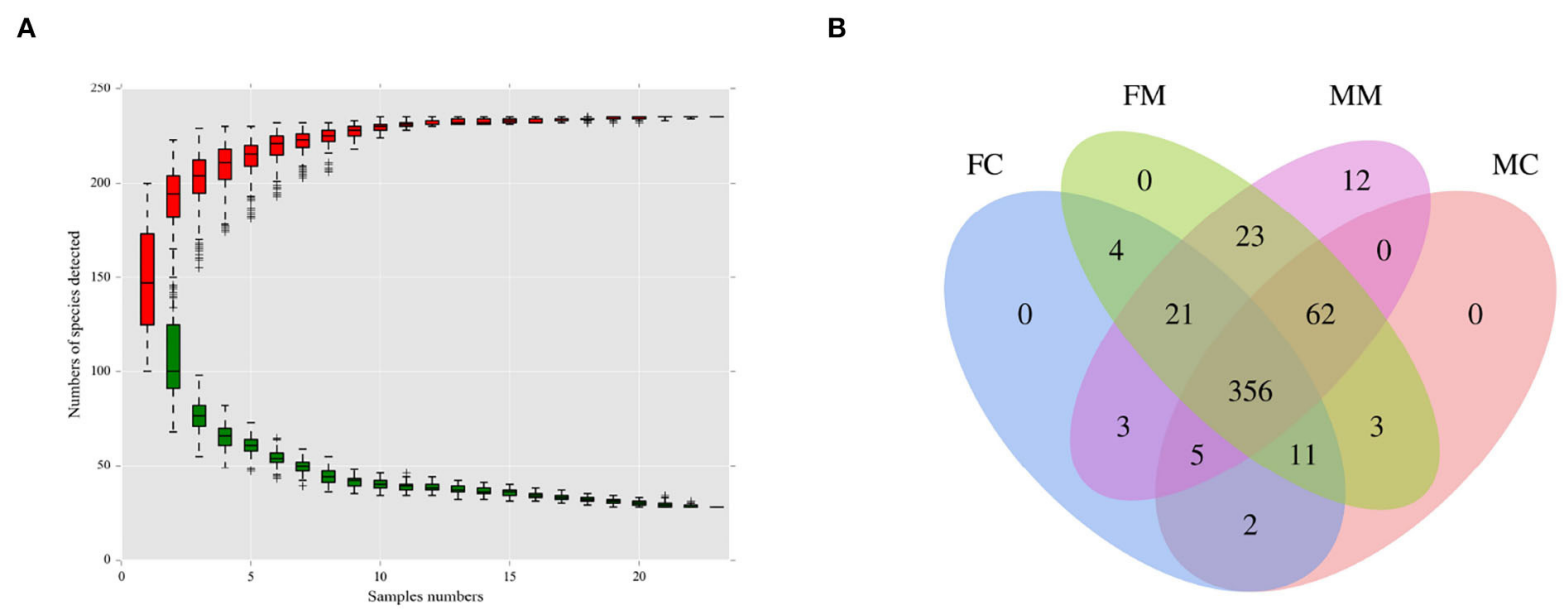
**(A)** The species accumulation curve was used to evaluate whether the sequencing volume was sufficient to cover all taxa and reflects the species richness of the intestinal microbiota. **(B)** Venn diagram of each group based on the number of OTUs. FC, luminal content of female mice; FM, mucosa of female mice; MM, mucosa of male mice; MC, luminal content of male mice.

As shown in Figure 1A, when the number of species approaches 230, the curve flattens, indicating that the number of species meets the analysis criteria. As shown in Figure 1B, the four groups had 356 shared OTUs; the MC and FC groups found 65 and 28 unique OTUs, respectively. MM and MC groups had 20 and 18 unique OTUs, respectively.

### Bacterial diversity analysis

**Figure 2:**
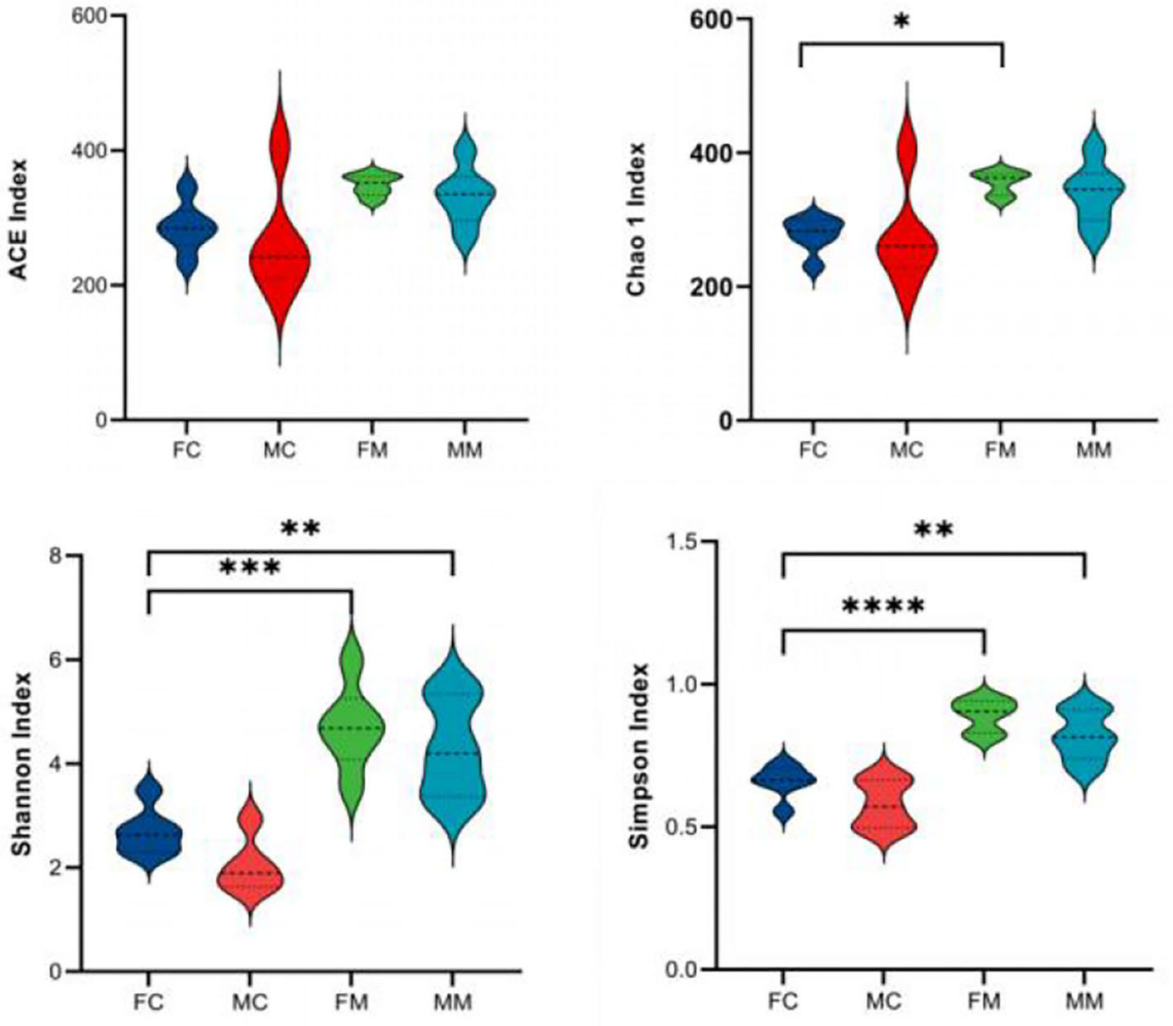
The α diversity index was calculated based on the OTU classification level, consisting of ACE, Chao 1, Shannon, and Simpson. *p < 0.05, **p < 0.01, ***p < 0.001, ****p < 0.0001.

α and β diversities explain the richness and diversity of microbial communities from different dimensions. α diversity refers to the richness and diversity of microbial communities and species within a living territory, expressed in four indicators: Chao 1, Shannon, ACE, and Simpson. As shown in Figure 2, there are no significant sex differences between LM and MM in the Chao 1, Shannon, ACE, and Simpson indices.

**Figure 3:**
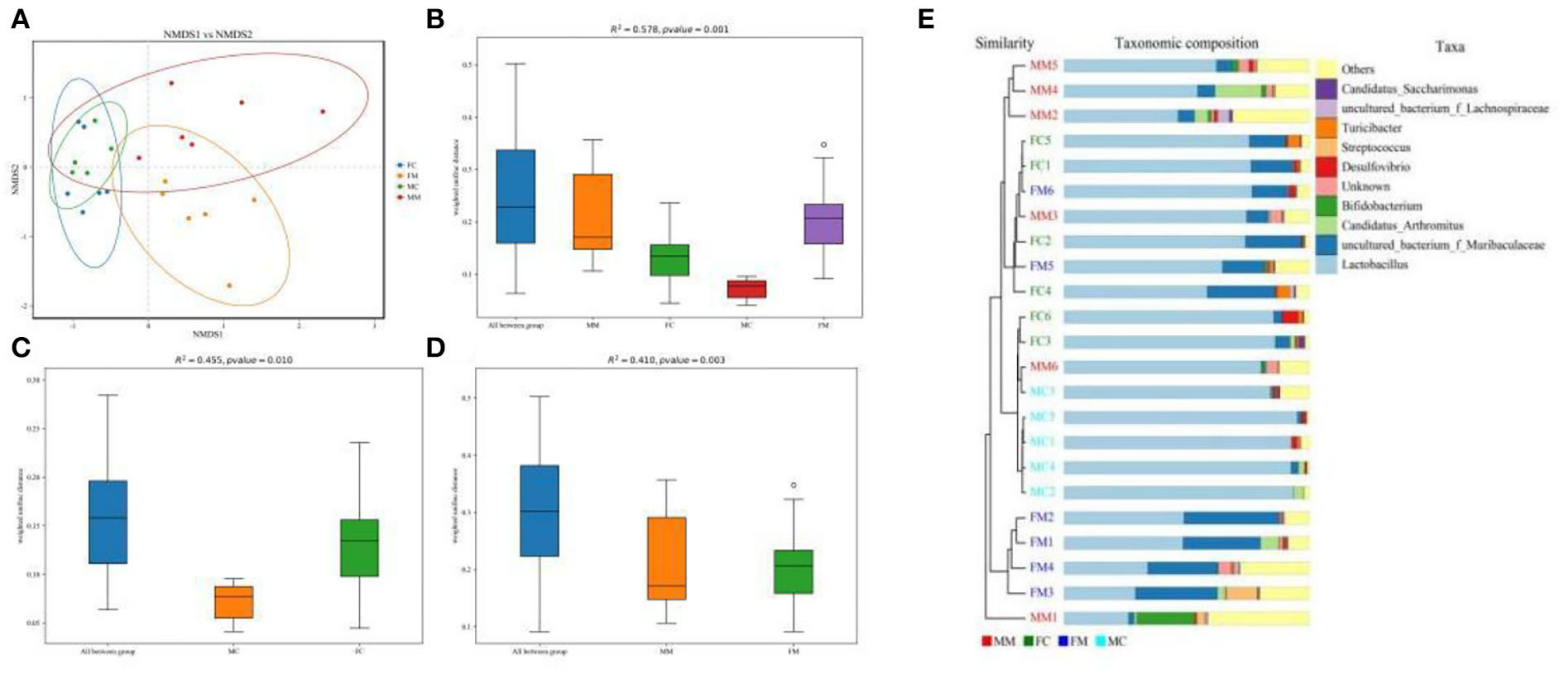
**(A)** Non-metric multidimensional scaling (NMDS) analysis (based on weighted UniFrac distance). Different groups of samples were colored differently. Distance between the dots presented a difference. NMDS with stress levels of less than 0.2 is acceptable. The closer the sample is to the coordinates, the higher the similarity. **(B–D)** Analysis of similarities (ANOSIM) (based on weighted UniFrac distance) can test for a significant difference in β-diversity among samples of different groups. The y-axis represents β-distance; the box above “All between *” represents the β-distance data of samples of all groups, while the box above “All within *” represents the β-distance data of samples within all groups. The closer the *R*-value is to 1, the difference between groups is higher than the difference within groups; the smaller the *R*-value is, the smaller difference between groups; and the value of *p* < 0.05 indicates high reliability of the test. **(E)** Combination of the phylogenetic tree and histogram based on the unweighted group average clustering method.

β diversity refers to the difference in the number and distribution of species in different environmental communities, reflecting not only the diversity distance between samples but also the degree of differentiation between bacterial communities. As shown in the NMDS analysis (Figure 3A), there was a small distance between the FM and MM groups (stress = 0.1365). The ANOSIM analysis (Figures 3B–D) showed significant differences between the FC and MC groups and the FM and MM groups (*p* < 0.01). The phylogenetic tree combined with a histogram of species distribution (Figure 3E) intuitively indicates subtle differences among groups, with the MC group being distinguishable from others.

### Bacterial composition analysis of intestinal microbiota

**Figure 4:**
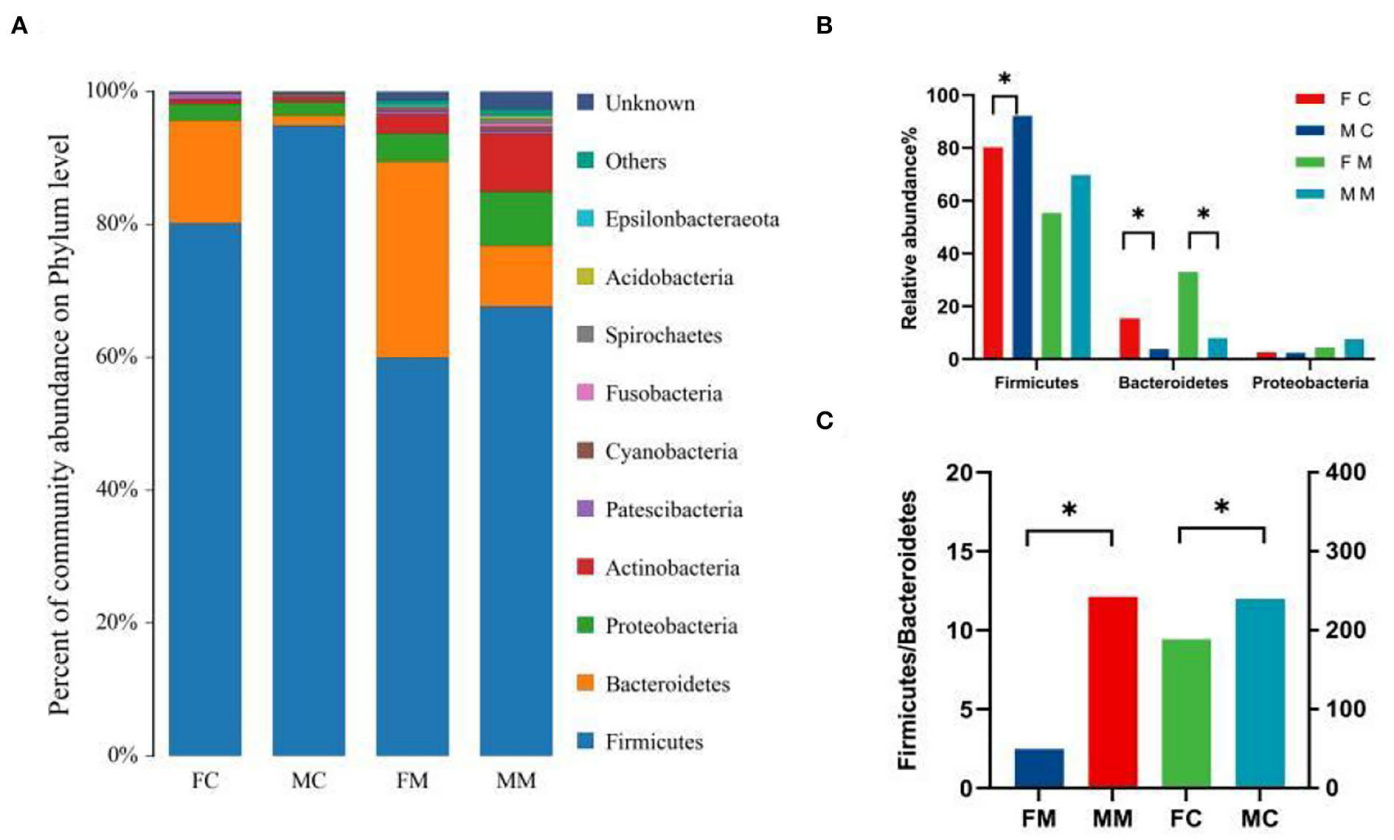
**(A)** The composition and relative abundance of species at the phylum level of each group, **(B)** the abundance of Bacteroidetes, Firmicutes, and Proteobacteria in each group (*p < 0.05), and **(C)** the Firmicutes/Bacteroidetes (F/B) ratio for each group.

To further investigate the differences in the intestinal microbiota of mice of different sexes, the relative abundance of microbial communities at the phylum and genus level in each group was counted. The combined abundance of Firmicutes, Bacteroidetes, Proteobacteria, and Actinobacteria exceeded 98% in LM (Figure 4A). The relative abundance of Firmicutes was higher in males than in females (*p* < 0.05, Figure 4B), while in the case of Bacteroidetes the opposite was true (*p* < 0.05, Figure 4B). The four phyla were also dominated in MAM, and the relative abundance of Bacteroidetes was also higher in females than in males (*p* < 0.05, Figure 4B). The Firmicutes/Bacteroidetes (F/B) ratio of MAM and LM was higher in males than in females (*p* < 0.05, *p* < 0.05, Figure 4C).

**Figure 5:**
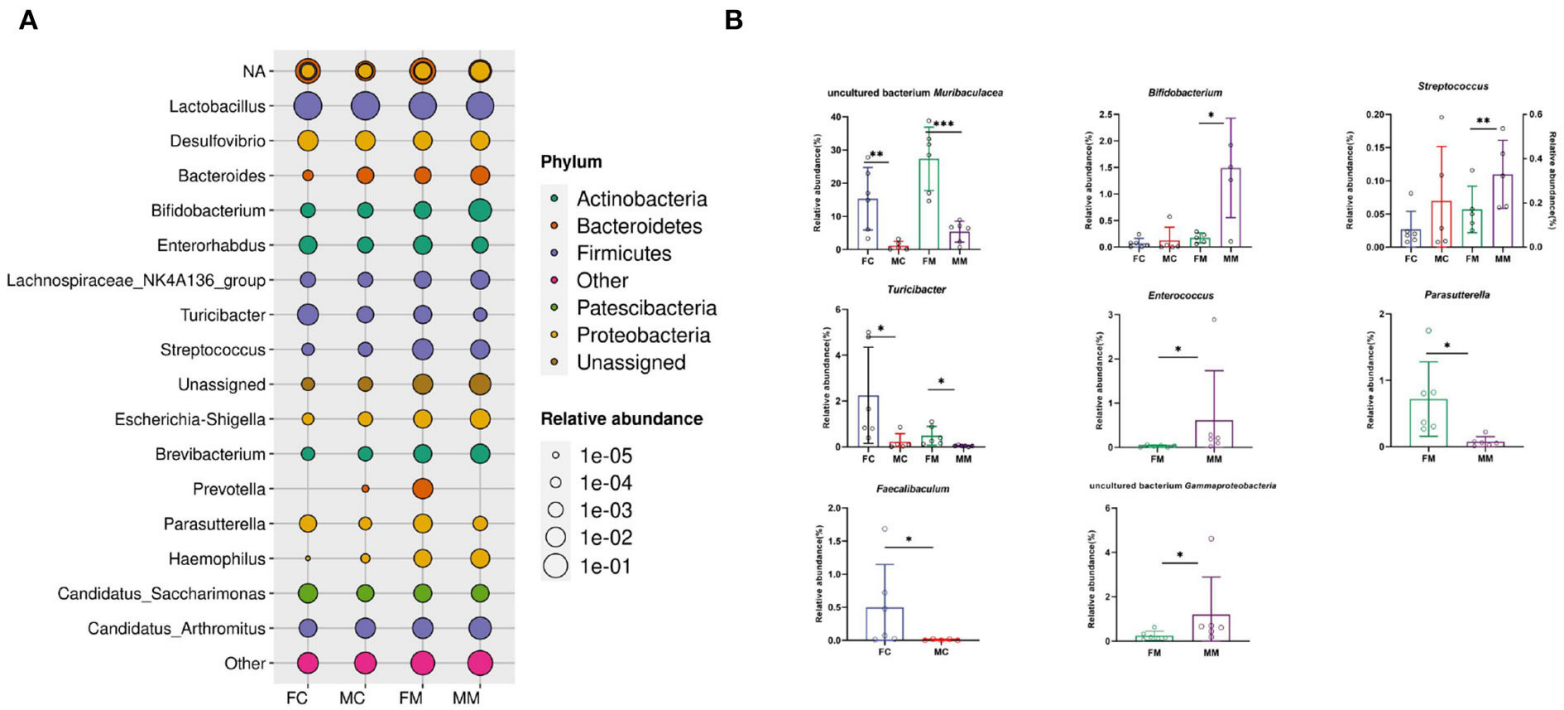
**(A)** The bacterial species and relative abundance of each group at the genus level, **(B)** illustration of sex differences in the relative abundance of the top 30 major genera (*p < 0.05, **p < 0.01, ***p < 0.01).

Figure 5 shows the relative abundance of each group of bacterial species at the genus level. The results showed that *Lactobacillus, Muribaculaceae, Candidatus Arthromitus, Bifidobacterium*, and *Desulfovibrio* were enriched, of which *Lactobacillus* had the highest relative abundance. The relative abundance of *Muribaculaceae, Turicibacter*, and *Faecalibaculum* was significantly higher in the FC group than in the MC group (*p* < 0.01, *p* < 0.05, *p* < 0.05). A total of seven of the top 30 relative abundance had a significant sex difference in MAM. Specifically, the relative abundance of *Muribaculaceae, Turicibacter*, and *Parasutterella* was significantly higher in the FM group than in the MM group (*p* < 0.001, *p* < 0.05, *p* < 0.05), while the relative abundance of *Bifidobacterium, Gammaproteobacteria, Enterococcus*, and *Streptococcus* was significantly higher in the MM group (*p* < 0.05) than in the FM group. Overall, sex differences were more prominent in MAM than LM.

### Intestinal differential bacterial species analysis in each group

**Figure 6:**
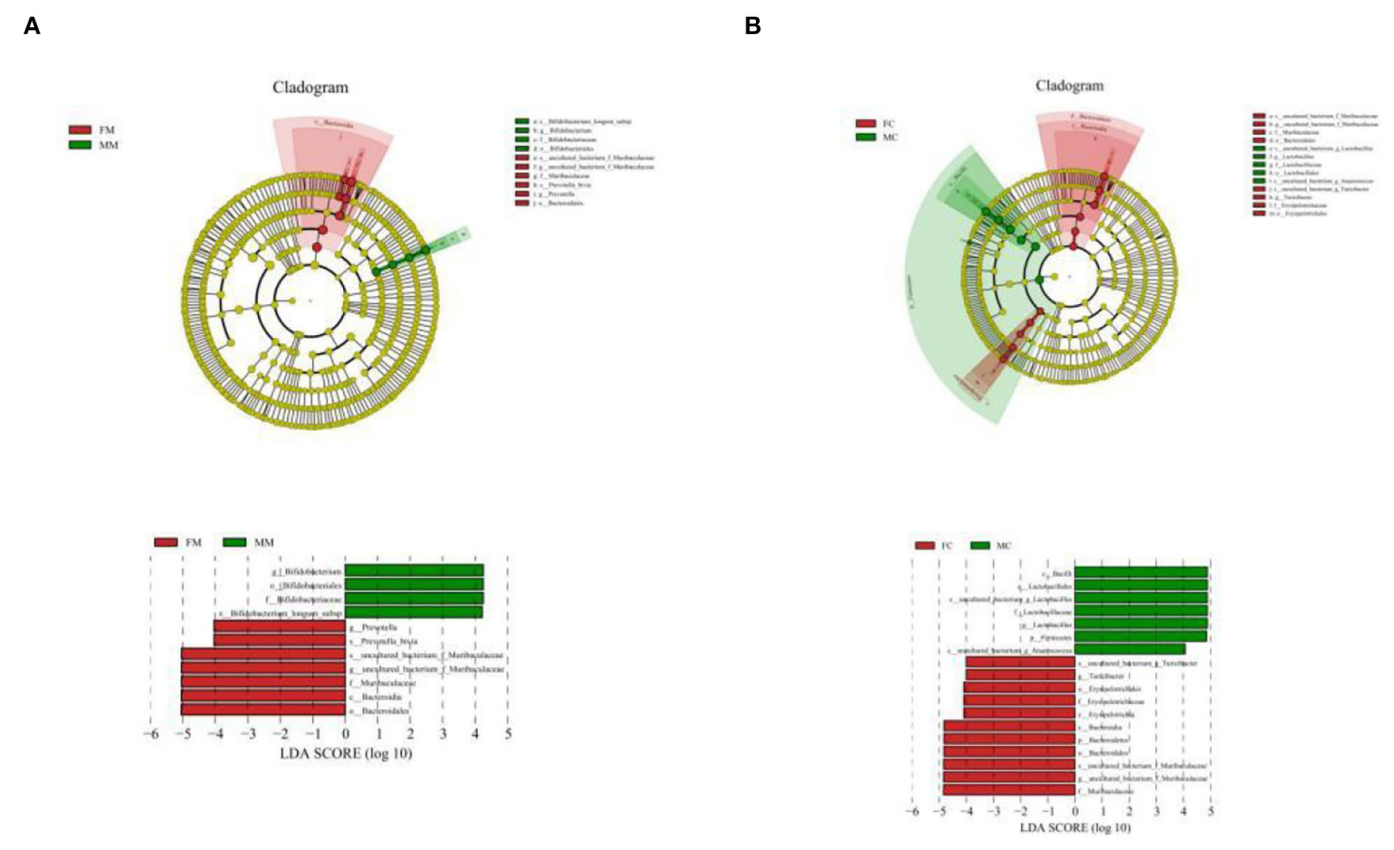
Cladogram based on the line discriminant analysis effect size (LEfSe) analysis and linear discriminant analysis (LDA) value distribution histogram of MAM **(A)** and LM **(B)**. In this figure, species with no significant differences are colored in yellow, and other colors stand for different groups. The LDA histogram showed the species whose LDA score is higher than the set value (the default is 4.0). The length of the histogram represents the impact of different species (i.e., LDA score), and different colors represent species in different groups. Different colors represent species in different groups, and nodes with different colors represent microbial groups that play an important role in the group represented by the color.

To further identify the species with the greatest differences in each group, we performed the LESef analysis (SCORE > 4, *p* < 0.05). The results (Figure 6A) showed that *Bifidobacterium longum* subsp. was a differential bacterium in the MC group, *Prevotella, Muribaculacea*, and *Bacteroidales* were the differential bacteria in the FC group. As shown in Figure 6B, *Lactobacillus* was a differential bacterium in the MM group and *Muribaculaceae, Erysipelotrichaceae, Turicibacter*, and *Anaerococcus* were the differential bacteria in the FM group.

**Figure 7:**
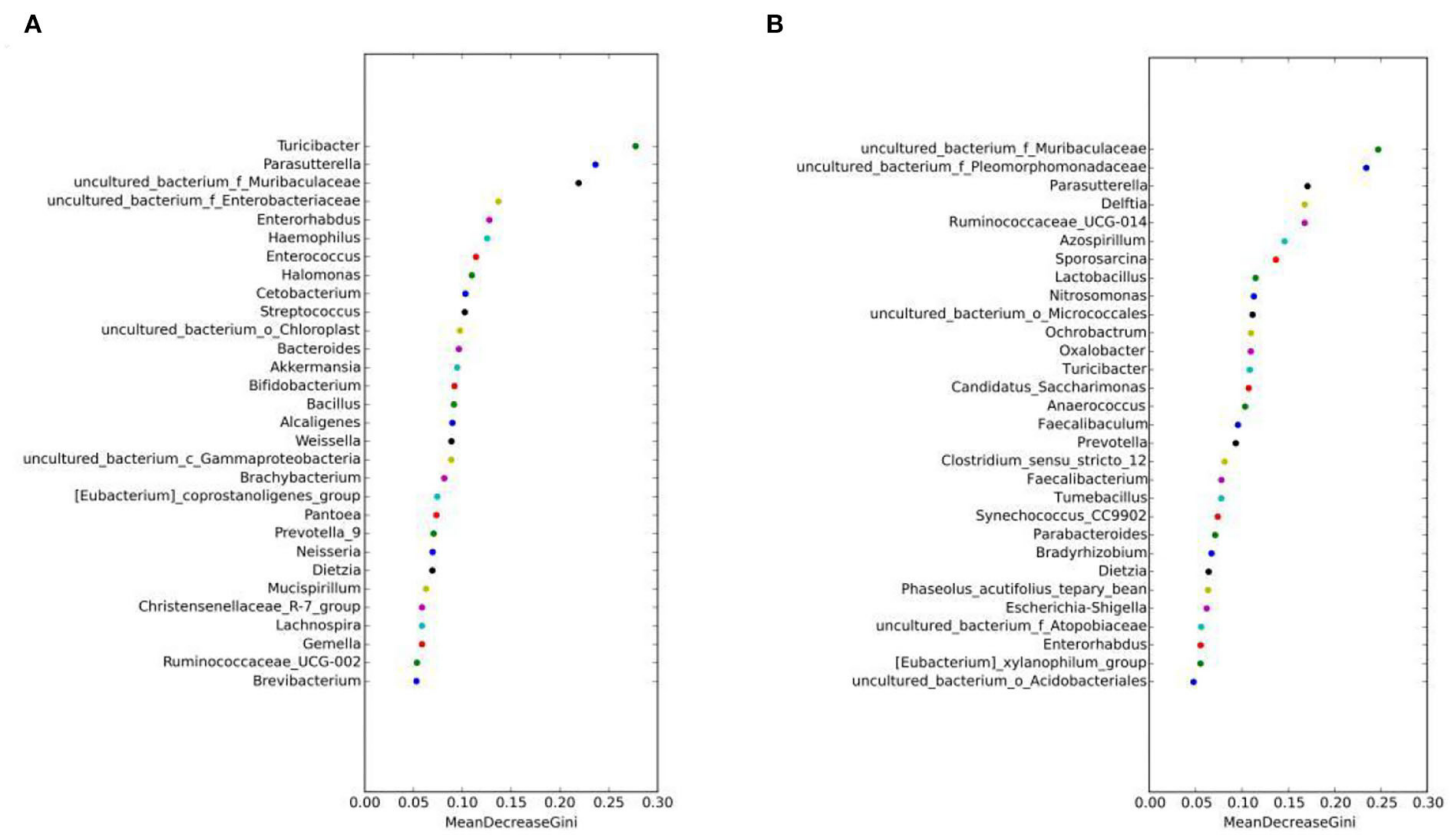
Random forest algorithm analysis of MAM **(A)** and intestinal LM **(B)**. The horizontal axis is the mean decrease Gini, and the vertical axis is the top 30 genera with the highest relative abundance. The larger the mean decrease Gini, the higher the contribution of bacteria.

The random forest algorithm analysis showed that *Parasutterella, Turicibacter*, and *Muribaculaceae* had the highest Gini index in the MAM group (Figure 7A), and *Muribaculaceae* and *Pleomorphomonadaceae* had the highest in the LM group (Figure 7B). In summary, *Parasutterella, Muribaculaceae*, and *Turicibacter* were the differential species identified in the FM and MM groups, and *Muribaculaceae* was the differential species identified in the FC and MC groups, which further confirmed that the sexual dimorphism of MAM was greater than that of LM.

### Correlation analysis of serum sex steroid hormones and the intestinal microbiota

**Figure 8:**
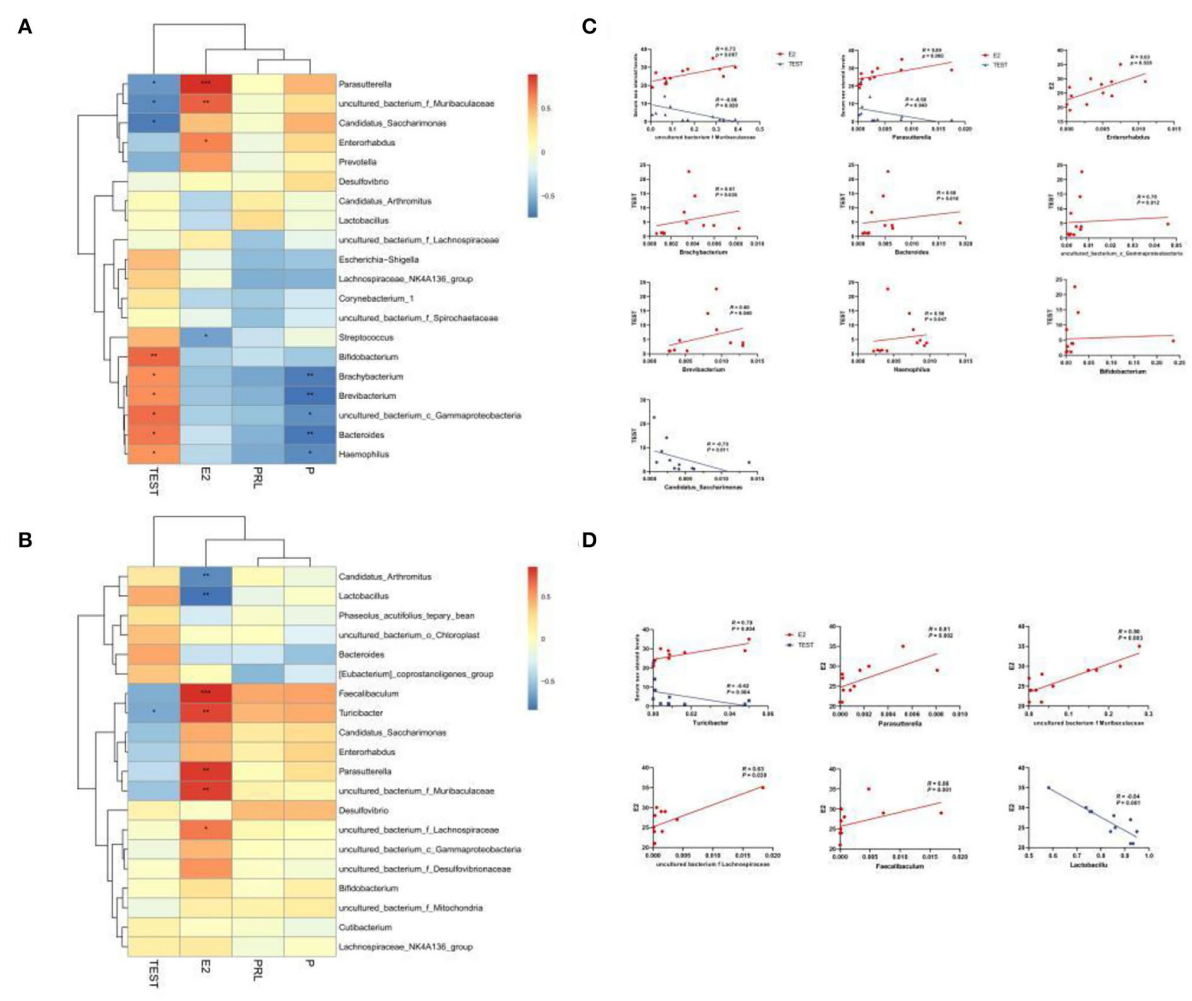
Heatmap of MAM **(A)** and LM **(B)** correlations with sex steroid levels. In the heat map, red represents a positive correlation, and blue represents a negative correlation; the closer to red, the closer the *R*-value is to 1; the closer to blue, the closer the *R*-value is to −1. In the linear correlation diagram of MAM **(C)** and LM **(D)**, the closer the *R*-value is to 1, the more obvious the correlation. *p < 0.05, **p < 0.01, ***p < 0.001.

To confirm whether the serum levels of sex hormones were related to the sexual dimorphism of the intestinal microbiota, we detected four serum sex hormone levels and found (Table 1) significant differences in serum levels of estradiol (E2) and T of male and female mice (*p* < 0.01). It could be seen in Figures 8A–C that, in MAM, *Parasutterella, Muribaculaceae, Enterorhabdus*, and E2 were significantly positively correlated (*p* < 0.05), especially since *Muribaculaceae* and *Parasutterella* had extremely significant differences (*R* > 0.073, *p* < 0.01; *R* > 0.089, *p* < 0.001). Moreover, *Brachybacterium, Bacteroides, Gammaproteobacteria, Brevibacterium*, and *Haemophilus* were positively correlated with T (*p* < 0.05), while *Candidatus Saccharimonas, Parasutterella*, and *Muribaculaceae* were negatively correlated with T (*p* < 0.05). In LM (Figures 8B,D), *Faecalibaculum, Turicibacter, Parasutterella, Muribaculaceae*, and *Lachnospiraceae* were positively correlated with E2 (*p* < 0.05), while *Lactobacillus* showed the opposite (*R* < −0.84, *p* ≤ 0.001) and *Turicibacter* was negatively correlated with T (*R* = −0.79, *p* < 0.05).

**Table 1:** Differences in serum sex steroid levels in mice ($\bar{X}$ ± *S, n* = 6).

**Sex steroid**	**E2 (Pg/mL)**	**Test (nmol/L)**	**Prolactin (ng/mL)**	**Progesterone (ng/mL)**
Female	29.33 ± 3.27	1.46 ± 0.72	234.83 ± 15.89	33.35 ± 11.13
Male	22.67 ± 2.88	9.62 ± 7.51	225.00 ± 15.30	22.14 ± 8.80
*P* value	0.004	0.004	0.326	0.093

### Functional prediction of MAM in mice of different sexes

To further explore whether the sexual dimorphism of MAM affects the potential function, we performed a functional prediction analysis of each group. As showed in Table 2, the glycosphingolipid biosynthesis—globo and isoglobo series is the only metabolic pathway with significant sex differences in MAM (*p* < 0.05).

**Table 2:** Functional prediction table of Kyoto Encyclopedia of Genes and Genomes (KEGG) level 3 of mucosa-associated microbiota (MAM).

**Level 1**	**Level 2**	**Level 3**	***P* value (adjusted)**
Metabolism	Lipid metabolism	Linoleic acid metabolism	0.614
		Sphingolipid metabolism	0.116
		Steroid hormone biosynthesis	0.293
		Biosynthesis of unsaturated fatty acids	0.584
	Glycan biosynthesis and metabolism	Glycosphingolipid biosynthesis - ganglio series	0.532
		Glycosphingolipid biosynthesis - globo and isoglobo series	0.022
		Glycosaminoglycan degradation	0.474
		Other glycan degradation	0.468
	Amino acid metabolism	Cyanoamino acid metabolism	0.302
		Selenocompound metabolism	0.514
	Metabolism of other amino acids	Alanine, aspartate and glutamate metabolism	0.527
	Energy metabolism	Photosynthesis - antenna proteins	0.613
Human diseases	Infectious diseases: Bacterial	Vibrio cholerae infection	0.458
Genetic information processing	Folding, sorting and degradation	Protein processing in endoplasmic reticulum	0.604
Environmental information processing	Membrane transport	ABC transporters	0.088
		Bacterial secretion system	0.609
	Transport and catabolism	Lysosome	0.465
Cellular processes	Cell growth and death	Apoptosis	0.628
	Cellular community - prokaryotes	Quorum sensing	0.547

## Discussion

### Differences in the characteristics of the intestinal microbiota in mice of different sexes

One of the important conclusions of this study is that MAM is more sex-sensitive than LM. As reported in the study, sex has a major influence on MAM (Borgo et al., 2018) than on LM, which is further confirmed in our experiment. First of all, β analysis showed sex differences only in the MAM. Secondly, among the top 30 bacteria with relative abundance at the genus level, the number of bacteria with sex differences in LM was 5, while the number of bacteria with sex differences in MAM was 7. Moreover, seven bacterial genera were associated with E2 secretion and only one with T secretion in LM, and four bacterial genera were associated with E2 secretion and nine with T secretion in MAM. In both physiology and pathology, MAM seems to be more susceptible to the host than to LM, which is attributed to the fact that MAM is less affected by food rather than LM. Zhang C. et al. (2021) proved that MAM is more susceptible to repeated stress-related diarrhea in comparison to LM. We conclude that MAM is more influenced by sex hormones, which can better represent sexual dimorphism. In further research, it is best to consider whether MAM is more suitable for the experiment. Unlike other studies, we did not find that the α diversity of female mice was higher than that of male mice, which was probably due to the different locations from which the samples were collected (Roager et al., 2016).

### The intestinal microbiota of mice with different sexes provides a new perspective on sexually dimorphic diseases

Our study found that the intestinal microbiota may be one of the important biological bases of sexual dimorphism in physiology and pathology. LEfSe and random forest algorithm analyses showed that *Muribaculaceae* and *Parasutterella* were the key genera to distinguish mice with different sexes. The relative abundance analysis of the intestinal microbiota revealed the highest concentration of *Muribaculaceae, Parasutterella*, and *Turicibacter* in the intestine of female mice. The correlation analysis of serum sex hormones with the intestinal microbiota illustrated that, in LM, both *Muribaculaceae* and *Parasutterella* were positively and negatively correlated with E2 and T, respectively, and *Turicibacter* was negatively correlated with T. All results demonstrate that *Muribaculaceae, Parasutterella*, and *Turicibacter* are the key species of intestinal sexual dimorphism.

*Parasutterella* may be involved in intestinal bile acid metabolism as the core intestinal microbiota of mice and humans (Ju et al., 2019). Bile acid synthesis rate and bile acid pool have been shown to be higher in females than in males (Turley et al., 1998). Related studies (Org et al., 2016) showed that bile acid metabolism changes significantly with hormonal changes, especially in high-fat and high-sugar diets. In our study, females of *Parasutterella* were highly abundant than male parasites, further suggesting that sex differences in bile acid metabolism might be related to intestinal *Parasutterella*. In addition, we found a positive correlation between the abundance of *Parasutterella* and E2, suggesting that sex hormones influenced bile acid metabolism in females by regulating the abundance of *Parasutterella*. Type 2 diabetes is a glucose metabolism disorder with significant sexual dimorphism; however, there is no clear mechanism to explain how this sex difference occurs. In animal studies, researchers proved that male mice have lower glucose tolerance compared to females, which is associated with intestinal microbiota and sex hormones (Gao et al., 2021). *Parasutterella* is a producer of succinate, which improves glucose homeostasis through intestinal gluconeogenesis (De Vadder et al., 2016; Canfora et al., 2019). Therefore, we hypothesize that sex hormones may be involved in the sexual dimorphism of glucose metabolism by regulating the abundance of *Parasutterella*.

Muribaculaceae is the dominant family in the intestine of mice. Studies showed that Muribaculaceae degrades dietary carbohydrates and rapidly adapts to carbohydrate-enriched diets to resist obesity (Obanda et al., 2018; Lagkouvardos et al., 2019). In our study, the abundance of *Bacteroides* and *Muribaculaceae* is significantly higher in females than in males and is positively and negatively correlated with E2 level and T, respectively. In addition, we found that the ratio of F/B (Jasirwan et al., 2021) used to evaluate the energy metabolism capacity was lower in female mice than in male mice (Figure 4C), which indicated that female mice had higher energy metabolism ability than male mice. As mentioned above, how sex hormones successfully and synthetically regulate the body's energy balance with *Muribaculaceae*, which will help to shed light on the formation mechanism of obesity and its prevalence in specific populations, is shown.

*Turicibacter* belonging to Firmicutes is involved in the metabolism of bile acids and cholesterol (Kemis et al., 2019). According to studies, *Turicibacter* may also be related to obesity, its abundance is positively correlated with high-density lipoproteins, and it may be involved in the formation mechanism of obesity by regulating cholesterol metabolism (Zheng et al., 2020). *Turicibacter* is therefore another important bacterium that reveals the biological basis of sex differences in obesity between males and females. There is a sex bias in mental disorders, which the sex-selective sex hormone theory seeks to uncover (Singh et al., 2021). Similarly, many studies demonstrated that the intestinal microbiota participates in the development of mental diseases through the brain-gut axis (Sharon et al., 2019; Qin et al., 2022). An interesting phenomenon is that males are more likely to exhibit the aggravation of autism symptoms with changes in their intestinal microbiota in times of adversity, but these have a limited impact on females (Rincel et al., 2019). In addition, the intestinal microbiota of male mice with autism showed a decrease in *Turicibacter*, and the lower the abundance of *Turicibacter*, the greater the social deficit (Szyszkowicz et al., 2017); however, the abundance of *Turicibacter* was reversed after intake of *Lactobacillus* (Kong et al., 2021). In our study, we further found that male mice had a significantly lower abundance of *Turicibacter* than female mice and were correlated with T. These results suggest that the development of autism in males may be related to congenital hormone levels and the intestinal microbiota, of which *Turicibacter* is worth exploring.

The abundance of *Bifidobacterium, Gammaproteobacteria*, and *Enterococcus* was significantly higher in the MM group than in the FM group and positively correlated with T, indicating that *Bifidobacterium, Gammaproteobacteria*, and *Enterococcus* were significantly regulated by sex hormones. Studies showed a marked increase in serum androgens and a lack of intestinal probiotics like *Bifidobacterium* in polycystic ovarian syndrome, but taking *Bifidobacterium* can reverse this trend. Unfortunately, the current experiment lacks evidence related to MAM (Zhang et al., 2019). The incidence of Crohn's disease is slightly higher in males than in females, but males had a milder disease severity than females. Studies confirm that this may be related to estrogen (Goodman et al., 2020). Studies showed that proteus plays a key role in the development of Crohn's disease by attacking the intestinal mucosa (Zhang J. et al., 2021). Other research also showed that *Gammaproteobacteria* are enriched within CD14^+^ macrophages from the intestinal lamina propria of patients with Crohn's disease vs. mucus (Sekido et al., 2020). However, in our research, *Proteus* has little correlation with estrogen, and its abundance is higher in the MM group than in the FM group. *Enterococcus* is a physiologically dominant species in the intestine of mice and humans, with strong tolerance and the ability to colonize the intestinal mucosa and inhibit pathogen damage to intestinal mucosal epithelial cells (O' Shea et al., 2012). So far, however, no link between *Enterococcus* and sex or sex hormones has been reported.

## Conclusion

Our study concluded that MAM is more sexually dimorphic than LM. We identified that various bacteria were prone to a certain sex and were highly correlated with E2 and T levels. *Parasutterella, Muribaculacea*, and *Turicibacter* are considered the best representatives of the intestinal microbiota associated with sex hormones. In addition, our study further found that *Parasutterella, Muribaculacea*, and *Turicibacter* are the key bacteria that cause intestinal sexual dimorphism. It is speculated that sex hormones may be involved in sexual dimorphism in bile acid metabolism by regulating the abundance of these bacteria. The dimorphism of *Turicibacter* in the intestinal microbiota also provides insight into how neurological diseases are more common in males than in females. Furthermore, our study also found that the glycosphingolipid metabolism of MAM had significant sex differences, which provided new clues for the mechanisms underlying sex differences in glucose metabolism.

However, our study only compared the differences in the intestinal microbiota in physiological mice and lacked experiments or data analysis for specific sex-associated diseases. Future studies should be based on open databases or more animal experiments to provide some reference for accurate clinical use.

## Data availability statement

The datasets presented in this study can be found in online repositories. The names of the repository/repositories and accession number(s) can be found below: NCBI, PRJNA847180.

## Ethics statement

The animal study was reviewed and approved by Animal Ethics Committee of Hunan University of Chinese Medicine. The number of the facility use permit: SYXK (Xiang) 2019-0009.

## Author contributions

YW: animal experiments, data analysis, and original draft writing. XP and XL: validation, review, and editing. DL: data analysis. ZT and RY: project administration and funding acquisition. All authors contributed to this article and approved the submitted version.

## Funding

This research was financially supported by the National Natural Science Foundation of China (Grant No. 81874460), the National Natural Science Foundation of China Regional Innovation and Development Joint Fund Key Support Project (Grant No. U21A20411), and the Provincial Natural Science Foundation of Hunan (Grant No. 2022JJ30440).

## Conflict of interest

The authors declare that the research was conducted in the absence of any commercial or financial relationships that could be construed as a potential conflict of interest.

## Publisher's note

All claims expressed in this article are solely those of the authors and do not necessarily represent those of their affiliated organizations, or those of the publisher, the editors and the reviewers. Any product that may be evaluated in this article, or claim that may be made by its manufacturer, is not guaranteed or endorsed by the publisher.
