# Supplementary material for: Sex hormones influence the intestinal microbiota composition in mice
Source: Front Microbiol. 2022 Oct 31;13:964847. doi: 10.3389/fmicb.2022.964847 (PMC9659915; doi:10.3389/fmicb.2022.964847)
Supplement: Supplementary file 1 [file Table_1.DOCX]

Supplementary Material

# Supplementary Data

| Accession | Sample Name | SPUID |  |
| --- | --- | --- | --- |
| SAMN28921309 | FC1 | FC1 | https://www.ncbi.nlm.nih.gov/biosample/28921309 |
| SAMN28921310 | FC2 | FC2 | https://www.ncbi.nlm.nih.gov/biosample/28921310 |
| SAMN28921311 | FC3 | FC3 | https://www.ncbi.nlm.nih.gov/biosample/28921311 |
| SAMN28921312 | FC4 | FC4 | https://www.ncbi.nlm.nih.gov/biosample/28921312 |
| SAMN28921313 | FC5 | FC5 | https://www.ncbi.nlm.nih.gov/biosample/28921313 |
| SAMN28921314 | FC6 | FC6 | https://www.ncbi.nlm.nih.gov/biosample/28921314 |
| SAMN28921315 | MC1 | MC1 | https://www.ncbi.nlm.nih.gov/biosample/28921315 |
| SAMN28921316 | MC2 | MC2 | https://www.ncbi.nlm.nih.gov/biosample/28921316 |
| SAMN28921317 | MC3 | MC3 | https://www.ncbi.nlm.nih.gov/biosample/28921317 |
| SAMN28921318 | MC4 | MC4 | https://www.ncbi.nlm.nih.gov/biosample/28921318 |
| SAMN28921319 | MC5 | MC5 | https://www.ncbi.nlm.nih.gov/biosample/28921319 |
| SAMN28921320 | FM1 | FM1 | https://www.ncbi.nlm.nih.gov/biosample/28921320 |
| SAMN28921321 | FM2 | FM2 | https://www.ncbi.nlm.nih.gov/biosample/28921321 |
| SAMN28921322 | FM3 | FM3 | https://www.ncbi.nlm.nih.gov/biosample/28921322 |
| SAMN28921323 | FM4 | FM4 | https://www.ncbi.nlm.nih.gov/biosample/28921323 |
| SAMN28921324 | FM5 | FM5 | https://www.ncbi.nlm.nih.gov/biosample/28921324 |
| SAMN28921325 | FM6 | FM6 | https://www.ncbi.nlm.nih.gov/biosample/28921325 |
| SAMN28921326 | MM1 | MM1 | https://www.ncbi.nlm.nih.gov/biosample/28921326 |
| SAMN28921327 | MM2 | MM2 | https://www.ncbi.nlm.nih.gov/biosample/28921327 |
| SAMN28921328 | MM3 | MM3 | https://www.ncbi.nlm.nih.gov/biosample/28921328 |
| SAMN28921329 | MM4 | MM4 | https://www.ncbi.nlm.nih.gov/biosample/28921329 |
| SAMN28921330 | MM5 | MM5 | https://www.ncbi.nlm.nih.gov/biosample/28921330 |
| SAMN28921331 | MM6 | MM6 | https://www.ncbi.nlm.nih.gov/biosample/28921331 |
